# Crystalline Defects Induced during MPCVD Lateral Homoepitaxial Diamond Growth

**DOI:** 10.3390/nano8100814

**Published:** 2018-10-10

**Authors:** Fernando Lloret, David Eon, Etienne Bustarret, Daniel Araujo

**Affiliations:** 1 Institute for Material Research, Hasselt University, 3590 Diepenbeek, Belgium; 2 IMOMEC (Instituut voor MateriaalOnderzoek in MicroElectronics), IMEC vzw, 3590 Diepenbeek, Belgium; 3 Dpto. Ciencia de los Materiales, Universidad de Cádiz, 11510 Puerto Real-Cádiz, Spain; daniel.araujo@uca.es; 4 Univ. Grenoble Alpes, CNRS (Centre National de la Recherche Scientifique), Institut NEEL, F-38042 Grenoble, France; David.Eon@neel.cnrs.fr (D.E.); etienne.bustarret@neel.cnrs.fr (E.B.)

**Keywords:** diamond, defects, MPCVD, TEM, dislocations, boron-doped diamond, lateral growth, selective growth

## Abstract

The development of new power devices taking full advantage of the potential of diamond has prompted the design of innovative 3D structures. This implies the overgrowth towards various crystallographic orientations. To understand the consequences of such growth geometries on the defects generation, a Transmission Electron Microscopy (TEM) study of overgrown, mesa-patterned, homoepitaxial, microwave-plasma-enhanced, chemical vapor deposition (MPCVD) diamond is presented. Samples have been grown under quite different conditions of doping and methane concentration in order to identify and distinguish the factors involved in the defects generation. TEM is used to reveal threading dislocations and planar defects. Sources of dislocation generation have been evidenced: (i) doping level versus growth plane, and (ii) methane concentration. The first source of dislocations was shown to generate <110> Burgers vector dislocations above a critical boron concentration, while the second induces <112> type Burgers vector above a critical methane/hydrogen molar ratio. The latter is attributed to partial dislocations whose origin is related to the dissociation of perfect ones by a Shockley process. This dissociation generated stacking faults that likely resulted in penetration twins, which were also observed on these samples. Lateral growth performed at low methane and boron content did not exhibit any dislocation.

## 1. Introduction

Defects in diamond are still one of the main restrictions for the development of commercial diamond electronics [1]. Indeed, homoepitaxial boron-doped layers usually contain planar and point defects [2,3,4,5] that have an undesirable impact on the resulting diamond-based device [1,6]. Many works have studied this topic in the case of 2D diamond growth [7,8,9] contributing to significantly improving the growth of diamond films and the control of the doping level to the point where the fabrication of δ-doped diamond layers could be contemplated [6,9,10,11,12,13]. Thanks to this progress, diamond electronic devices have been shown to be capable of operating within electronic circuits [6,12,13,14,15,16], even though these devices still suffer from shortcomings such as high electric field regions, low breakdown voltages, or high reverse current values. The design of alternative structures will allow to overcome these difficulties [17]. In this direction, overgrowth on mesa-etched substrate is being used to manage local doping in order to design three dimensional devices [18,19], as well as to improve the local surface roughness of homoepitaxial diamond films [20,21], or to reduce the density of threading dislocations. Unfortunately, many efforts are still required to reach the crystal quality required by electronic applications. Indeed, the study by Transmission Electron Microscopy (TEM) of the lateral diamond growth over 3D patterns has pointed out many lattice-related defects on the studied samples that can be attributed to lattice strain at the corners of trenches and mesa rectangle structures, or to additional surface effects [18,22]. In addition, heavy boron doping has also been shown to favor the generation of dislocations, even in nm-thick layers [2,3,8]. A better understanding and control of all factors generating defects is essential for the successful fabrication of diamond-based devices. To identify the crucial parameters that should be controlled to avoid such defects, and to understand the mechanisms responsible for the generation of dislocations, seven samples were grown on mesa-etched substrates under different conditions of pressure, temperature, doping and/or methane concentration (see Table 1), and studied by TEM in diffraction contrast mode. 

## 2. Materials and Methods 

**Figure 1:**
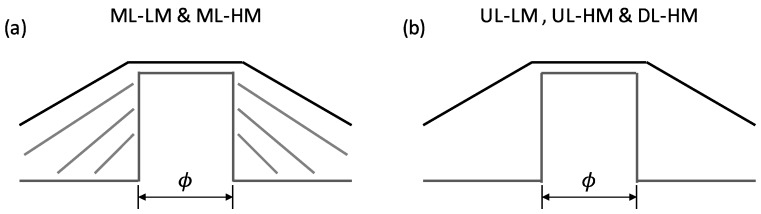
Schematics of samples grown. (**a**) Schema of a Ø-diameter disk multilayered grown corresponding to samples ML-LM and ML-HM where grey layers represent doped layers; (**b**) Schematics of a Ø-diameter disk single-layered grown corresponding to samples UL-LM, ML-HM and DL-HM.

Electronics grade, {001}-oriented, high pressure-high temperature (HPHT) diamond substrates have been etched by inductively coupled plasma reactive ion etching (ICP-RIE) using pure O_2_ gas, leaving a set of 0.8/1 µm height mesa-shaped cylindrical patterns. The process followed for the etching is described in [22]. Samples were overgrown by microwave plasma-enhanced chemical vapor deposition (MPCVD) in a NIRIM type reactor. Pressure was set at 33 Torr, and the temperature used during growth was ~900 °C. Methane was used as a gas precursor for diamond growth, and diborane for boron doping. Due to the use of the same reactor for every sample, on some occasions, oxygen was added to ensure the non-doping of the undoped layers. Table 1 shows the molar gas ratios used for each sample, as well as the rest of conditions. There, time is shown as growth minutes per layer. According to their growth conditions, samples can be grouped in: (i) multilayer doped/undoped, ML, (#A, #C and #E), undoped monolayer, UL, (#B, #D and #F), and doped monolayer, DL, (#G); or (ii) growth under high methane conditions, HM, (#C, #D, #E, #F and #G) and low methane concentration conditions, and LM (#A and #B). Such configurations are schematized in Figure 1.

The study was performed by transmission electron microscopy (TEM) using the 200 KV accelerating voltage microscopes FEG JEOL 2010 and Philips CM200 (Thermo Fisher, Hillsboro, OR, USA), and a 120 KV JEOL 1200FX microscope (JEOL (Europe) BV, Nieuw-Vennep, The Netherlands). Electron-transparent lamellas for the TEM observations were obtained by lift-out method along the radius of the initial cylindrical patterns (dubbed “disks” hereafter) by a focused ion beam (FIB) with a scanning electron microscope (SEM) dual beam FEI Quanta 200 3D (Thermo Fisher, Hillsboro, OR, USA) [23].

## 3. Results

**Figure 2:**
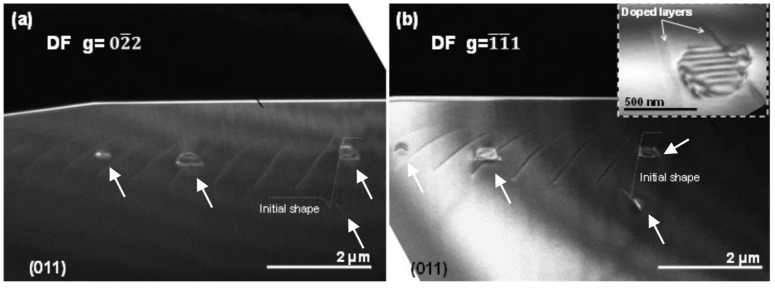
Dark field micrographs of sample #A recorded under two beam conditions oriented at the {011} pole using the (**a**) $\left[0\bar{2}2\right]$ and (**b**) $\left[\bar{1}\bar{1}1\right]$ reflections. Inset shows one of the planar defects in detail.

Figure 2 shows dark-field micrographs of sample #A (ML-LM) taken under two beam conditions oriented along the {011} pole. Figure 2a shows a dark field micrograph of part of one of the overgrown disks, recorded using the $\left[0\bar{2}2\right]$ reflection. The initial radial profile of the disk pattern, i.e., the cylindrical structure generated after the ICP step and before the lateral MPCVD growth, is marked by a dashed white line. Doped layers are visible by a soft white contrast revealing the growth orientations [22], and a residual contrast shows three planar defects (see arrows). Figure 2b shows a dark-field micrograph of the same region recorded using the $\left[\bar{1}\bar{1}1\right]$ reflection. The initial radial profile has also been marked here in the same way, and doped layers are visible, albeit with dark contrast. Planar defects are more contrasted under these TEM conditions, and four of them are visible in the region of interest (one was practically not visible using the $\left[0\bar{2}2\right]$ reflection). They are the only defects observed, and they were generated near or in the doped layers. Note that two of these planar defects lie in the substrate, and one of them (the one which is invisible in Figure 2a) in the bottom corner of the initial disk. The inset of Figure 2b shows one of these defects at a higher magnification. However, the same study was performed on sample #B (UL-LM), grown under the same conditions as #A, but as a single undoped layer. It shows the same type of planar defect in cross-section view. This fact dismisses the influence of the thin doped layers, used as markers (TEM contrast) to follow the growing planes in the generation of the planar defects observed on these samples.

**Figure 3:**
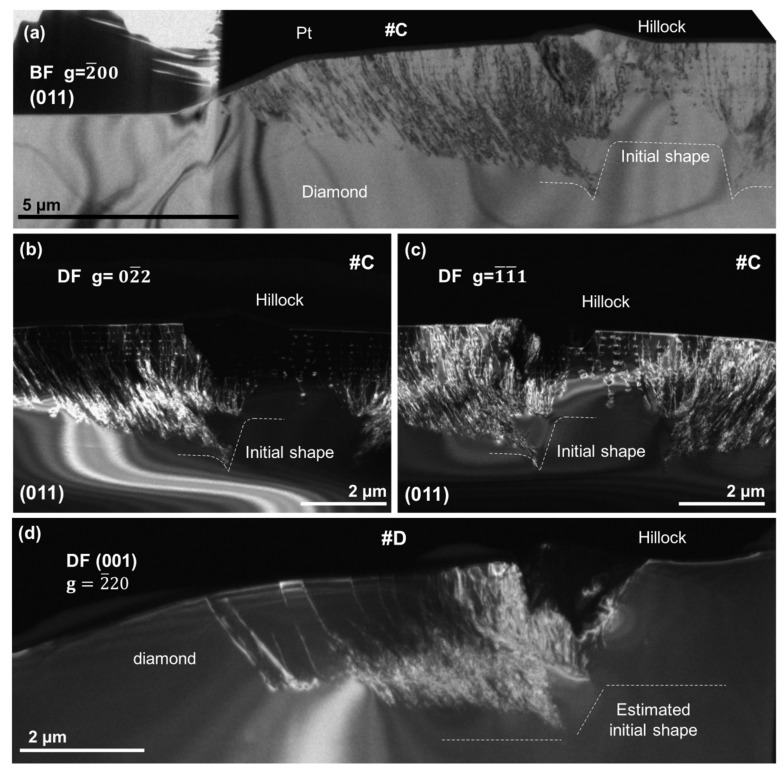
Micrographs of sample #C recorded under two beam conditions along the (011) pole. White dashed lines mark the initial shape of the etched substrate. (**a**) Bright-field micrograph recorded using the $\bar{2}00$ reflection. Platinum marked by Pt is due to the FIB-lamella preparation. The superficial defect observed on the top of the sample and generated by penetration twins was labelled “Hillock”. Dislocations are clearly visible as dark contrasts; (**b**) Dark-field micrograph recorded using the $0\bar{2}2$ reflection. Most dislocations are not visible because the lamella is tilted; (**c**) Dark-field micrograph recorded using the $\bar{1}\bar{1}1$ reflection. Dislocations are shown as white contrasts; (**d**) DF micrograph of sample #D in two beam conditions oriented along the (001) pole recorded using the $\bar{2}20$ reflection. Dashed white line marks the location of the initial disk. Dislocations are observed to come from both corners of the initial disk in the same way as was observed for sample #C.

Sample #C (ML-HM) is analogous to sample #A, but grown at a higher methane concentration. Figure 3a shows a bright-field micrograph of a broader view of this sample, and (b) and (c) two dark field micrographs of the same region of this sample. Micrographs have been recorded under two beam conditions along the {011} pole. In contrast to samples #A and #B, sample #C involved many linear defects. Most of these defects originate from the corners of the initial disk, highlighted by a white dashed line on the micrographs of Figure 3, and they bend their trajectories following the growth orientation.

The invisibility criterion ($\vec{g}\cdot \vec{b}=0$) has been used in order to determine the Burgers vector of the observed dislocations. Because dislocations are mostly visible by using reflections, (a) $g=\bar{2}00$ and (c) $g=\bar{1}\bar{1}1$ and remained invisible with the reflection (b) $g=0\bar{2}2$, a Burgers vector $b=\frac{1}{6}\left[211\right]$ has been determined. There is still some remaining contrast in Figure 3b. Such contrast is attributable to two main factors: (i) interaction between dislocations that can form additional kinds of dislocations by recombination, and (ii) that $b=\frac{1}{6}\left[211\right]$ dislocations are not fully invisible at $g=0\bar{2}2$ since the lamella is a bit tilted. This is also the reason why additional white contrasts are shown. When large lamellas are polished down to very low thicknesses, inner tensions bend them, making it impossible to have the whole lamella oriented along the same zone axis, thereby generating the mentioned contrast artifact.

In addition to the dislocations, there is an inhomogeneity on the top of the upper corner, shown by a dark contrast in Figure 3b,c. This contrast reveals a different orientation of the region with respect to the rest of the sample. This is attributed to penetration twins that generate hillocks on the top surface [24,25,26,27].

Figure 3d shows dark field micrographs of sample #D (UL-HM) taken under two beam conditions oriented along the (001) pole using the $\bar{2}20$ reflection. The dislocations are in a similar configuration to those of sample #C. Most of them are coming from the corners of the estimated initial profile of the disk, with the same $b=\frac{1}{6}\left[211\right]$ Burgers vector. Hillocks are also clearly observed on this sample as regions of different contrast. Overall, the defects are not significantly different in both samples.

It is known that methane concentration directly affects the overgrowth orientations on patterned substrates [28,29]. Here, from Figure 2 and Figure 3, it is shown that this methane concentration also has an important influence on defect generation. For low methane concentrations, only a few planar defects were observed, whereas samples grown at higher concentrations showed a high density of threading dislocations. In contrast, in this doping range, boron addition did not have any effect.

**Figure 4:**
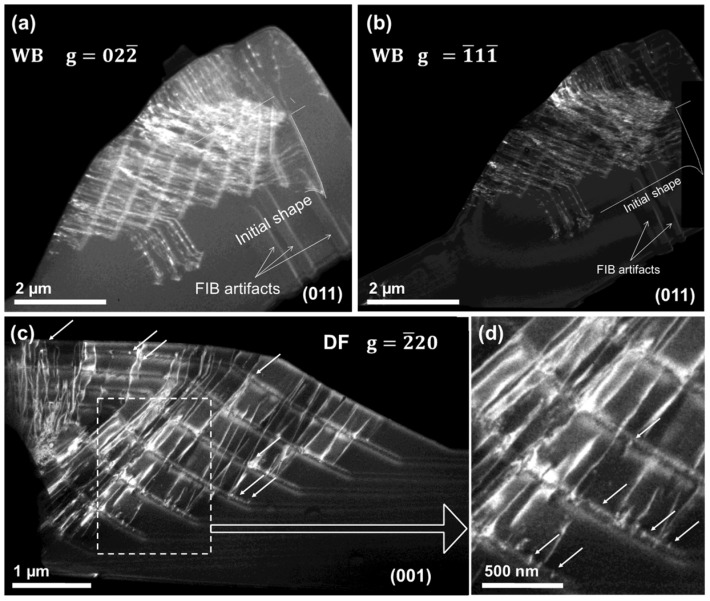
Weak beam (WB) micrographs of sample #E recorded along the (011) pole using the $\left[02\bar{2}\right]$ (**a**) and $\left[\bar{1}1\bar{1}\right]$ (**b**) reflections. Dark field (DF) micrographs of the same sample #E on the (001) pole recorded with the $\left[\bar{2}20\right]$ reflection (**c**), and a magnified region of it (**d**). Arrows point out tipped dislocations in (**c**), enlarged in (**d**).

Sample #E (ML-HM), grown at high methane concentration, but highly boron-doped, is analyzed in order to determine the influence of higher boron doping levels. Figure 4 shows weak beam micrographs of a lamella recorded at the $\left[02\bar{2}\right]$ (a) and $\left[\bar{1}1\bar{1}\right]$ (b) reflections of the {011} pole, and a second lamella oriented along the pole {001} recorded at the $\left[\bar{2}20\right]$ reflection ((c) and (d)). Micrographs have been recorded in diffraction-contrast mode and two beam conditions considering three different reflections to determine the Burgers vectors. This sample, analogously to the previously-observed samples #C and #D, also shows a high density of dislocations. However, most of the TEM-contrast produced by the dislocations in the micrographs seems to originate in the doped layers instead of at the corners of the initial disk. This is clearly observable in the Figure 4a,b, micrographs that have been used to determine the Burgers vector of the threading dislocations according to the invisibility criterion. They correspond to $b=\frac{1}{2}\left[01\bar{1}\right]$ and $b=\frac{1}{2}\left[0\bar{1}1\right]$. The origin of such defects on the doped layer was confirmed in Figure 4c and, at higher magnification, Figure 4d. There, dislocations were clearly coming from the doped layers, and climb up to the surface of the sample following the growth orientation. Moreover, Figure 4d shows point-shaped defects, here called tipped defects, which are only visible with the $\left[\bar{2}20\right]$ reflection. They are marked with white arrows and correspond to dislocations that usually are observed at the p^++^/undoped interface. These dislocations were generated in the growth plane, and were probably induced by boron-boron proximity effects, as already reported for heavily boron-doped layers [3]. Thus, first the dislocation lies in the growth plane, and then, at some point, it can bend to follow the growth direction. Tipped defects are shown to be located inside the layers. Some of them have been marked by white arrows in Figure 4c for clarity. From all these micrographs, doped layers seem to be the main region where dislocations are generated. In fact, they were all generated in the (111) plane of growth or near orientations that have been reported to yield lower critical boron levels (CBL) than (100) [3].

**Figure 5:**
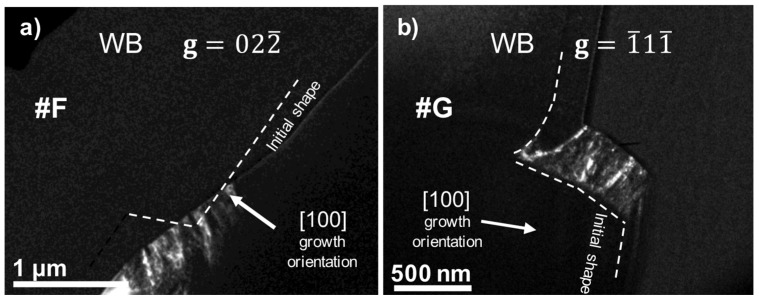
(**a**) Weak beam micrograph of sample #F oriented along the <011> direction, recorded under two beam conditions using $g=02\bar{2}$ reflection; (**b**) Weak beam micrograph of sample #G using the **g** = $\bar{1}1\bar{1}$ reflection. In both cases, dislocations appear as white contrasts. A dashed white line traces the tentative initial disk.

Figure 5a shows a weak beam micrograph of sample #F (UL-HM), grown at a high methane concentration, recorded in two beam conditions with the sample oriented along the (011) pole. There was not any compositional contrast between the substrate and the overgrowth region, since sample #F was grown as a single undoped layer. However, defects tentatively mark the shape of the initial disk, facilitating the identification of the top and bottom corners. Invisibility criterion has been employed again, determining a $b=\frac{1}{6}\left[21\bar{1}\right]$ Burgers vector.

Figure 5b shows a weak beam micrograph of sample #G (DL-HM) corresponding to an overgrown boron doped diamond layer. This sample involves {111} lateral growth direction, as shown by the micrographs. However, dislocations were not observed with the **g** = $\bar{2}00$ reflection. Using the invisibility criterion, the assigned Burgers vector is $b=\frac{1}{2}\left[01\bar{1}\right]$. Defects are not generated in any specific positions of the disk, such as at the corners, but they seem to be originated at different places, and to cover the whole epilayer.

Both samples, #F and #G, grown at high methane concentration, display a lateral side with many dislocations covering the layer growth more are less uniformly. However, the Burge’s vector of each sample is different, being $b=\frac{1}{6}\left[21\bar{1}\right]$ for the undoped sample, #F, and $b=\frac{1}{2}\left[01\bar{1}\right]$ for the doped one, #G.

Planar defects were also found in the samples. Stacking faults observed on samples #C, #D, and #E were shown to correspond to the ∑3 coincident-site-lattice (CSL) structure and {111} type [30].

## 4. Discussion

Table 2 summarizes the results presented so far. The first evidence is that, in terms of defects, sample #A and #C have the same behavior as #B and #D samples, respectively. This implies that very thin and lightly boron-doped layers do not have any influence on defect formation, and that the four samples are undoped-like. In addition, samples #A and #B are free of linear defects, and only planar defects were generated during their growth.

Samples #C and #D, similar to the undoped sample #F, showed threading dislocations with the same $b=\frac{1}{6}\langle 211\rangle$ Burgers vector. In contrast, highly doped samples (samples #E and #G) exhibit a high density of threading dislocations with $b=\frac{1}{2}\langle 011\rangle$ Burgers vector. According to this observation, in undoped samples dislocations with $b=\frac{1}{6}\langle 211\rangle$, Burgers vectors are generated, whereas dislocations in doped samples are associated with $b=\frac{1}{2}\langle 011\rangle$ Burgers vectors.

In previous works [3,29], the effect of the doping per growth orientation in terms of dislocation density was analyzed. There, a graphic with dislocation density versus the boron content per [111] and [100] growth orientations was shown. The results extracted from this graph agree with the ones observed here. Alegre et al. [3] reported a critical boron doping level, CBL, in diamond samples depending on the CH_4_/H_2_ molar ratio and on the growth directions. From this study, for a percentage of methane similar to that used for samples #C, #E, and #G, (0.5%), the CBL is 6.5 × 10^20^ atm/cm^3^ and 3.2 × 10^21^ atm/cm^3^ for <111> and <100> growth directions, respectively. Along the <111> direction, the CBL was of the same order as for samples #C and #G (estimated bellow 1 × 10^21^ atm/cm^3^), and higher for the <100> growth orientation. However, considering a homogenous distribution of boron atoms independently of the growth direction, these levels are smaller than the doping level of the sample #D, which is 1.2 × 10^21^ atm/cm^3^ for the <111> growth direction, and very close to the limit for <100>. This is consistent with the fact that most of the threading dislocations (TDs) were generated along the lateral (111) growth plane, and that the density of dislocations was almost zero for the (100) plane.

The existence of two families of Burgers vectors on the samples was shown to be related with the boron concentration: doped samples show $b=\frac{1}{2}\langle 011\rangle$ threading dislocations. On the other hand, growth parameters logically play an important role. High growth rates (i.e., high methane concentrations) induce the generation of dislocations with $b=\frac{1}{6}\langle 211\rangle$ Burgers vectors. The $b=\frac{1}{2}\langle 011\rangle$ Burgers vector corresponds to perfect dislocations which move the atoms to identical sites within the crystal. $b=\frac{1}{6}\langle 211\rangle$ Burgers vector, in contrast, corresponds to partial dislocations that generate additional stacking faults (SF). This explains the fact that SFs are observed in the samples, together with $b=\frac{1}{6}\langle 211\rangle$ dislocations. Moreover, perfect dislocations can be dissociated into partial ones when such a division is energetically favorable. This is the case of $b=\frac{1}{2}\langle 011\rangle$ dislocations, which may be divided in two $b=\frac{1}{6}\langle 211\rangle$ ones in the so-called Shockley partial dislocation basis. From these results, we can conclude that the growth mechanism has generated $b=\frac{1}{2}\langle 011\rangle$ perfect TDs. On undoped or slightly doped samples, these perfect dislocations were dissociated by the Shockley process. This process changes the Burgers vector family and introduces SFs. These SFs may form micro {111} disoriented planes that favor the generation of penetration twins, such as those observed on samples #C and #D.

In fact, in some cases, the density of dislocations is too high (see Figure 3). This favors their annihilation or even their recombination to form additional dislocations (as for example the Thompson Stair-Rod: **b** = 1/6<110>, the Aeure Stair-Rod **b** = 1/3<110> and/or the Obtuse Stair-Rod **b** = 1/3<100> or **b** = 1/6<310>).

When a sample is highly doped, such as, for example, sample #E, dislocations are blocked and the perfect $b=\frac{1}{2}\langle 011\rangle$ TDs can’t be dissociated. This is the reason why sample #E and #G do not show any partial dislocation.

## 5. Conclusions

This work identified low methane concentration as the best condition to achieve crystal quality, since only planar defects were observed. In addition, it showed that lateral growth at methane concentrations equal to or higher than 0.5% resulted in the generation of the $b=\frac{1}{2}\langle 011\rangle$ family of TDs. Such TDs tend to dissociate into partial $b=\frac{1}{6}\langle 211\rangle$ TDs by a Shockley mechanism, generating SFs in turn that likely result in penetration twins. However, when samples are highly doped, boron atoms block the dislocations, avoiding their dissociation. Nevertheless, this high boron atom concentration itself acted as a defect generator due to the local stress it introduced. As was expected from previous studies [3,29], the critical boron doping level depended on the plane of growth, resulting in different densities of dislocations within the same sample, depending on the growth sector.

## Figures and Tables

**Table 1:** Conditions used during the growth of each sample.

Sample	Layer	CH_4_/H_2_	O_2_/H_2_	B_2_H_6_/CH_4_	Time (min)	Group
#A	Doped	0.25%	-	10,700	2 × 13	ML-LM
	Undoped	0.1%	-	-	60 × 13	
#B	Undoped	0.1%	-	-	840 × 1	UL-LM
#C	Doped	0.5%	-	9600	1 × 13	ML-HM
	Undoped	0.75%	0.32%	-	10 × 13	
#D	Undoped	0.75%	0.32%	-	140 × 1	UL-HM
#E	Doped	0.5%	-	14,000	11 × 10	ML-HM
	Undoped	0.75%	0.32%	-	11 × 10	
#F	Undoped	0.75%	0.25%	-	30 × 1	UL-HM
#G	Doped	0.5%	-	6000	10 × 1	DL-HM

**Table 2:** Summary of Burgers vectors of the dislocations present in each sample.

Sample	Layer	Burgers Vector
#A	Low Doping-Low Methane	No
	Undoped-Low Methane	
#B	Undoped-Low Methane	No
#C	Low Doping-High Methane	$b=\frac{1}{6}\langle 211\rangle$
	Undoped-High Methane	
#D	Undoped-High Methane	$b=\frac{1}{6}\langle 211\rangle$
#E	High Doping-High Methane	$b=\frac{1}{2}\langle 011\rangle$
	Undoped-High Methane	
#F	Undoped-High Methane	$b=\frac{1}{6}\langle 211\rangle$
#G	High Doping-High Methane	$b=\frac{1}{2}\langle 011\rangle$
